# Inhibition of NOX2 or NLRP3 inflammasome prevents cardiac remote ischemic preconditioning

**DOI:** 10.3389/fphys.2023.1327402

**Published:** 2024-01-15

**Authors:** Sandra Benavides, Rodrigo Palavecino, Jaime A. Riquelme, Luis Montecinos, José Pablo Finkelstein, Paulina Donoso, Gina Sánchez

**Affiliations:** ^1^ Physiopathology Program, Institute of Biomedical Sciences, School of Medicine, Universidad de Chile, Santiago, Chile; ^2^ Advanced Center for Chronic Diseases (ACCDiS), Faculty of Chemical and Pharmaceutical Sciences & Faculty of Medicine, Universidad de Chile, Santiago, Chile; ^3^ Interuniversity Center for Healthy Aging, Santiago, Chile; ^4^ Physiology Program, Institute of Biomedical Sciences, School of Medicine, Universidad de Chile, Santiago, Chile

**Keywords:** NOX2, NLRP3 inflammasome, heart, ischemia-reperfusion, infarct size, remote ischemic preconditioning

## Abstract

**Introduction:** Short episodes of ischemia-reperfusion (IR) in the heart (classical ischemic preconditioning, IPC) or in a limb (remote ischemic preconditioning, RIPC) before a prolonged ischemic episode, reduce the size of the infarct. It is unknown whether IPC and RIPC share common mechanisms of protection. Animals KO for NOX2, a superoxide-producing enzyme, or KO for NLRP3, a protein component of inflammasome, are not protected by IPC. The aim of this study was to investigate if NOX2 or NLRP3 inflammasome are involved in the protection induced by RIPC.

**Methods:** We preconditioned rats using 4 × 5 min periods of IR in the limb with or without a NOX2 inhibitor (apocynin) or an NLRP3 inhibitor (Bay117082). In isolated hearts, we measured the infarct size after 30 min of ischemia and 60 min of reperfusion. In hearts from preconditioned rats we measured the activity of NOX2; the mRNA of Nrf2, gamma-glutamylcysteine ligase, glutathione dehydrogenase, thioredoxin reductase and sulfiredoxin by RT-qPCR; the content of glutathione; the activation of the NLRP3 inflammasome and the content of IL-1β and IL-10 in cardiac tissue. In exosomes isolated from plasma, we quantified NOX2 activity.

**Results:** The infarct size after IR decreased from 40% in controls to 9% of the heart volume after RIPC. This protective effect was lost in the presence of both inhibitors. RIPC increased NOX2 activity in the heart and exosomes, as indicated by the increased association of p47phox to the membrane and by the increased oxidation rate of NADPH. RIPC also increased the mRNA of Nrf2 and antioxidant enzymes. Also, RIPC increased the content of glutathione and the GSH/GSSG ratio. The inflammasome proteins NLRP3, procaspase-1, and caspase-1 were all increased in the hearts of RIPC rats. At the end of RIPC protocol, IL-1β increased in plasma but decreased in cardiac tissue. At the same time, IL-10 did not change in cardiac tissue but increased by 70% during the next 50 min of perfusion.

**Conclusion:** RIPC activates NOX2 which upregulates the heart’s antioxidant defenses and activates the NLRP3 inflammasome which stimulates a cardiac anti-inflammatory response. These changes may underlie the decrease in the infarct size induced by RIPC.

## 1 Introduction

Ischemic heart disease and acute myocardial infarction constitute a major cause of death worldwide. Following a myocardial infarction patients are at increased risk of heart failure, arrhythmias, and death (Yellon and Hausenloy, 2007). Although reperfusion is mandatory following cardiac ischemia, reperfusion itself increases the damage and the infarct size (Vanden Hoek et al., 1996; Yellon and Hausenloy, 2007) and several approaches have been developed to reduce the damaging effects of IR. Classical ischemic preconditioning is a strategy whereby brief episodes of ischemia by occlusion of a coronary artery followed by reperfusion, activate endogenous protective mechanisms and decrease IR damage after a lethal ischemia (Murry et al., 1986). Protection also occurs when the brief preconditioning ischemic episodes do not occur in the heart but in a distant tissue, such as a limb (Kharbanda et al., 2002). This is called remote ischemic preconditioning. Preclinical studies demonstrate that the brief episodes of ischemia in the limb trigger neuronal and humoral signals, which activate protective mechanisms in the heart (Hausenloy and Yellon, 2008; Lim et al., 2010). It is not completely understood how these protective mechanisms are initiated. Still, conceivably, the first line of defense should be against the molecules or pathways that initiate IR damage.

Reactive oxygen species and inflammation contribute significantly to the harmful effects of ischemia-reperfusion (Granger and Kvietys, 2015; Toldo and Abbate, 2018), yet, if they are inhibited during preconditioning, protection does not develop. In preclinical studies, KO animals for NADPH oxidase type 2 (NOX2), an enzyme that oxidizes NADPH to generate superoxide anion, lose the ability to develop protection against ischemia-reperfusion injury when subjected to classical ischemic preconditioning (Bell et al., 2005). Tachycardia or exercise-induced preconditioning are also prevented by inhibition of NOX2 (Sánchez et al., 2008). Likewise, KO animals for the NLRP3 inflammasome, a complex involved in innate immunity, are not protected by classical ischemic preconditioning (Zuurbier et al., 2012).

There is scarce information about the involvement of NOX2 or NLRP3 inflammasome in remote ischemic preconditioning, which theoretically activates other pathways of protection (Pearce et al., 2021). The aim of this work is to evaluate if the activity of NOX2 and the NLRP3 inflammasome is also involved in the protection produced by RIPC. Knowing the molecular mechanisms of cardiac protection may help improve the design and application of protective protocols in patients exposed to ischemia-reperfusion injury.

## 2 Materials and methods

This study conformed to the Guide for the Care and Use of Laboratory Animals, published by the U.S. National Institutes of Health (NIH, Publication No. 85-23, revised in 1996), and was approved by the Institutional Ethics Review Committee, protocol number CBA 22547-MED-UCH.

### 2.1 Remote ischemic preconditioning protocol

Rats (male, 250–300 g) were anesthetized (80 mg/kg pentobarbital,i.p.) and subjected to four cycles of 5 min ischemia and 5 min reperfusion using a tourniquet placed on the right hind limb. Controls were maintained for an equivalent period of time under anesthesia. When used, Apocynin (5 mg/kg, i.p.) or Bay11-7082 (130 μg/kg, i.p.) were administered 5 or 30 min respectively, before starting RIPC. An adjustable heating pad kept the body temperature constant at 37°C. Following RIPC, some hearts were rapidly frozen under liquid nitrogen and kept at −80°C until analyzed. Other hearts, were perfused in a Langendorff system to measure the infarct size. In these animals, a sternotomy was performed and heparin 100 U/kg IV was administered. The heart was rapidly excised, mounted in a temperature-regulated heart chamber, and perfused retrogradely via the ascending aorta using a peristaltic infusion pump at a constant flow of 10–14 mL/min to generate an initial mean coronary (aortic) perfusion pressure (CPP) of 60–70 mm Hg with physiological modified Krebs Henseleit Buffer solution containing (in mM) NaCl (128.3), KCl (4.7), CaCl2 (1.35), NaHCO3 (20.2), NaH2PO4 (0.4), MgSO4 (1.1), glucose (11.1), pH 7.4 at 37 °C when equilibrated with a mixture of 95% O2/5% CO2. Perfusion solution and bath temperatures were maintained at 37°C by a thermostatically controlled water circulator. We measured the left ventricular pressure developed by the heart by a latex balloon inserted in the left ventricle, connected to a pressure transducer (Bridge Amp ML221 AD Instruments, Australia), and filled with normal saline to produce a left ventricle end-diastolic pressure of 5–10 mm Hg. Hearts were paced at 240 beats/min. After 20 min stabilization, hearts were subjected to 30 min of global ischemia followed by 60 min of reperfusion.

### 2.2 Measurement of the infarct size

Hearts subjected to IR in the absence (IR, N = 15; RIPC, N = 7) or in the presence of inhibitors (Apocynin, N = 4 and Bay11-7082, N = 5) were perfused with triphenyl tetrazolium chloride (TTC, Sigma-Aldrich, St. Louis, MO) to measure the infarct size as described previously (Vila-Petroff et al., 2007).

### 2.3 Preparation of tissue fractions

Whole-ventricle homogenates and membrane-enriched fractions were prepared from frozen tissue, as described before (Donoso et al., 2014; Sanchez et al., 2016). Proteins were determined in western blots as described below.

### 2.4 Preparation of exosomes

At the end of the preconditioning protocol, blood samples were collected in sodium citrate blood collecting tubes (Becton Dickinson) from the abdominal aorta. Blood (5 mL) was centrifuged at 2,000 x g for 10 min at room temperature. The plasma obtained was centrifuged at 10,000 x g for 30 min, and the supernatant was carefully separated (platelet-free plasma) and diluted with an equal volume of PBS. The diluted plasma was ultracentrifuged at 100,000xg for 70 min. The pellet was washed once with PBS and finally resuspended in 100 μL of PBS and kept frozen at −80°C. Exosomes were characterized using a Nanosight LM10-HS (Nanosight Ltd., Malvern, United Kingdom).

### 2.5 RNA Isolation and qRT-PCR

Total RNA was isolated from cardiac tissue using TRIzolTM (Thermo Fisher, Waltham, MA, United States) according to the manufacturer’s instructions. RNA from each sample was used for reverse transcription (RT) using the iScriptTM cDNA synthesis kit (Bio-Rad). The cDNA was used for quantitative polymerase chain reaction (qPCR) analysis in an amplification system (CFX96 Touch, Bio-Rad, Hercules, CA, United States) using Brilliant III Ultra-Fast SYBR^®^ Green QPCR Master Mix (Agilent, Santa Clara, CA, United States). Values were normalized to the expression of the constitutive 18S gene. The 2^−ΔΔCT^ method was used to calculate relative transcript abundances. Primers used for RT-PCR were as follows: Nrf2: Forgard 5′-GCC AGC TGA ACT CCT TAG AC -3’; Reverse 5′-GAT TCG TGC ACA GCA GCA -3. γ-GCL (gamma-glutamylcysteine ligase): Forward 5′-ATC​TGG​ATG​ATG​CCA​ACG​AGT​C-3’; Reverse 5′-CCT​CCA​TTG​GTC​GGA​ACT​CTA​CT-3’. Glutathione Reductase: Forward 5′-ACC​ACG​AGG​AAG​ACG​AAA​TG-3’; Reverse 5′-ATC​TCA​TCG​CAG​CCA​ATC​C-3’. Thioredoxin Reductase: Forward 5′-GGT​GAA​CAC​ATG​GAA​GAG​CA-3’; Reverse 5′-GGA​CTT​AGC​GGT​CAC​CTT​GA-3’. Sulfiredoxin-1: Forward 5′-CCC​AAG​GCG​GTG​ACT​ACT​A-3′; Reverse 5′-GGC​AGG​AAT​GGT​CTC​TCT​CT-3′. 18S: Forward 5′-AAA​CGG​CTA​CCA​CAT​CCA​A-3’; Reverse 5′-CCT​CCA​ATG​GAT​CCT​CGT​TA-3’

### 2.6 Glutathione content

Total glutathione (GSH + GSSG) concentration was determined in heart homogenates as described by Griffith (1980).

### 2.7 Interleukin-1B determination

IL-1B was determined by ELISA using a commercial kit from Invitrogen (catalog number BMS 630), according to the manufacturer’s instructions

### 2.8 Interleukin-10 determination

IL-10 was determined in heart homogenates by ELISA using a commercial kit from Invitrogen (catalog number BMS 629) according to the manufacturer’s instructions

### 2.9 Caspase-1 activity

Caspase activity was determined using a commercial kit from Abcam (abcam 39412) according to manufacturer’s instructions

### 2.10 Western blot analysis

Proteins were separated by electrophoresis in polyacrylamide gel (8 or 15% gels), transferred to PVDF membranes (Bio-Rad), and immunoblotted. The primary antibodies used were: anti-p47phox (SAB 4502810, Sigma Aldrich), anti-gp91phox (Sc-74514, Santa Cruz), anti-GAPDH (G9545, Sigma Aldrich), anti-CD81 (555675, BD Biosciences), anti-CD63 (Sc-15363, Santa Cruz), anti-caspase-1 (Sc-56036, Santa Cruz), anti-NLRP3 (MA5-32255, Thermo Fisher), anti-ASC (Sc-271054, Santa Cruz). Antigen-antibody reactions were detected by ECL (Amersham Biosciences), and blots were quantified using ImageJ Lab software. Results were normalized to GADPH.

### 2.11 Determination of protein concentration

Total protein concentration was determined using the bicinchoninic acid assay (Pierce BCA Protein Assay Kit; Thermo Scientific, Rockford, IL).

## 3 Results

### 3.1 Remote ischemic preconditioning and infarct size

**FIGURE 1:**
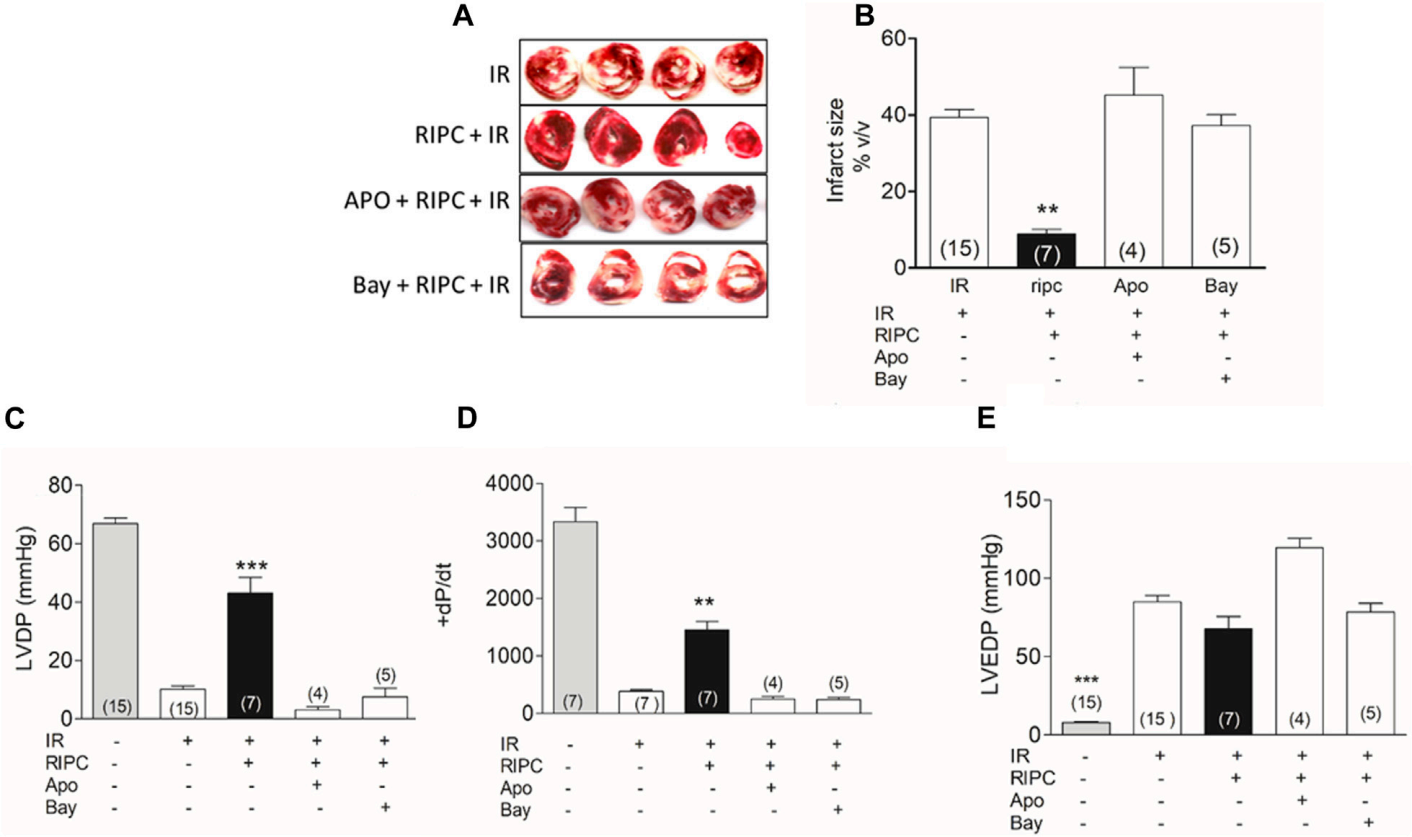
Inhibition of NOX2 or NLRP3 inflammasome prevent the protection by RIPC. **(A)** Representative hearts slices after IR. **(B)** Quantification of infarct size in hearts as those shown in A. **(C)** left ventricle developed pressure (LVDP). **(D)** +dP/dt. **(E)** Left ventricular end diastolic pressure (LVEDP). IR: hearts subjected to 30 min of ischemia and 60 min of reperfusion; RIPC: hearts subjected to remote ischemic preconditioning prior to the IR protocol; Apo: apocynin, Bay: Bay11-7082; Mean ± SEM; Number in the bar correspond to the experimental N; Kruskal-Wallis test followed by Dunn’s multiple comparison test; ***p < 0.001 and **p < 0.01 RIPC vs. all conditions.

Global ischemia resulted in an average infarct of 40% of the heart volume in isolated rat hearts. Hearts from remote ischemic preconditioned (RIPC) rats had significantly smaller infarct sizes, averaging 9% of the heart (Figures 1A, B). When apocynin, a NOX2 inhibitor, and ROS scavenger, or BAY11-7082, an NLRP3 inflammasome inhibitor, were given before RIPC, the protective effect was eliminated (Figures 1A, B). RIPC also improved the recovery of the pressure developed by the heart after ischemia (Figure 1C). Hearts subjected to 30 min of ischemia and 60 min of reperfusion developed a pressure of less than 10 mm Hg while control hearts developed over 65 mmHg after being perfused in Langendorff during an equivalent period of time (110 min). Hearts from RIPC rats developed a pressure of 43 mm Hg on average, a value that is 65% of the pressure developed by controls (Figure 1C). The developed pressure after IR was negligible when apocynin or BAY11-7082 was given before RIPC (Figure 1C). Changes in +dP/dt followed the pattern of LVDP (Figure 1D) but left ventricular end diastolic pressure (LVDEP) remained elevated in all cases (Figure 1E). These results suggest that RIPC prevents irreversible damage caused by IR, but persist a transitory mechanical dysfunction that will require more time to improve. Importantly, the elimination of ROS or inhibition of the NLRP3 inflammasome causes the loss of the protection on the irreversible damage afforded by RIPC.

### 3.2 RIPC activates NOX2 in the heart

**FIGURE 2:**
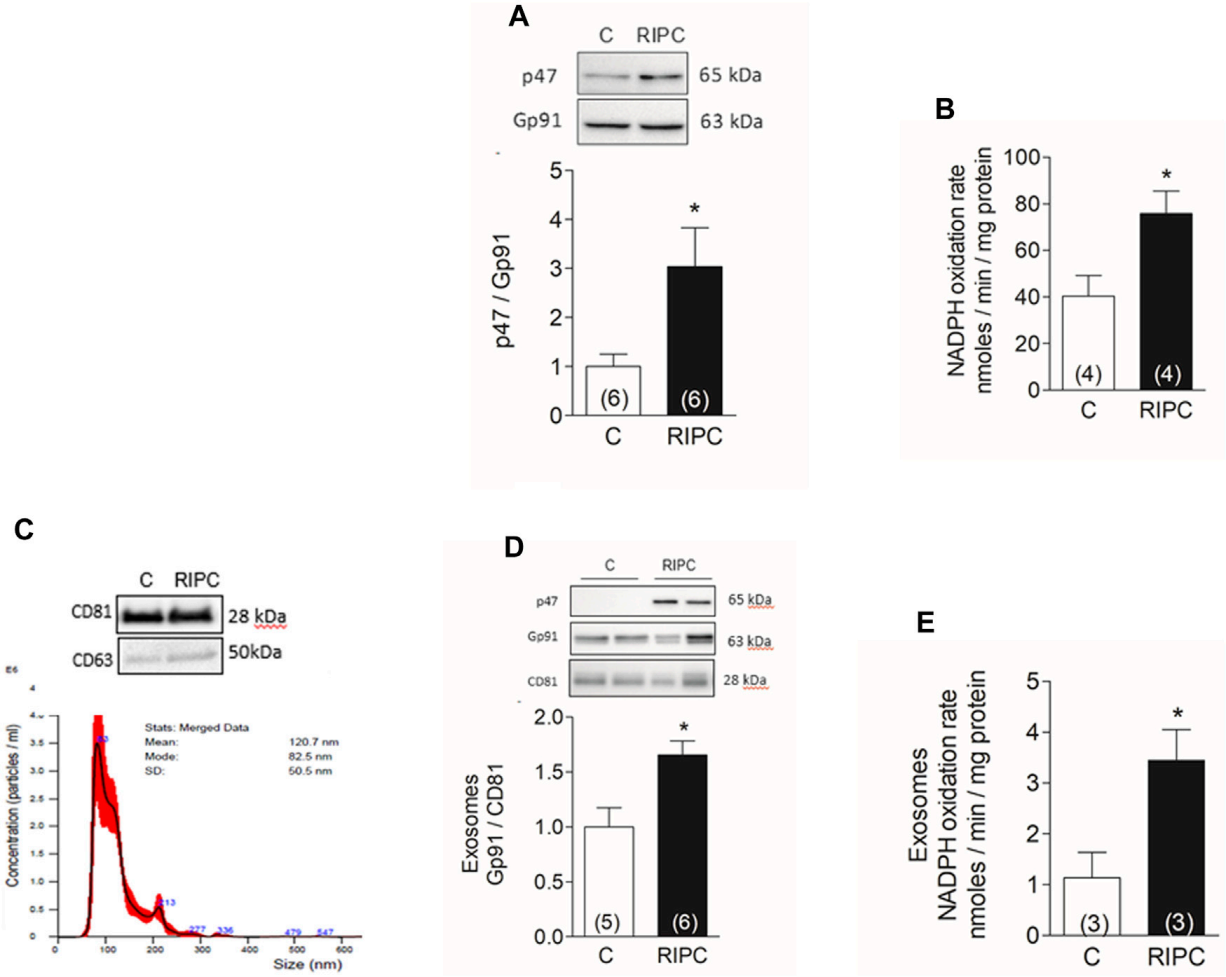
RIPC activates NOX2 in the heart and exosomes: **(A)** Representative western blots and quantification of NOX2 subunits p47phox and gp91phox in heart homogenates. **(B)** NADPH oxidation rate in membrane-enriched fraction isolated from hearts. **(C)** representative Western blot of marker proteins CD63 and CD81 in exosomes (top) and nanoparticle tracking analysis of particle concentration vs. size; the solid line represents the mean, and the standard deviation is shown in red (bottom). **(D)** representative Western blot and quantification of gp91phox normalized to CD81 in exosomes. **(E)** NADPH oxidation rate in exosomes. Bars represent the mean ± SEM; the number of hearts or exosome preparations analyzed is shown in the bar. Mann Whitney test; *p < 0.05.

The effect of apocynin and BAY11-7082 on the infarct size suggests that ROS and the NLRP3 inflammasome are both involved in the protection by RIPC. We and others have determined before that NOX2, a superoxide-generating enzyme, is critical in the protection induced by classical ischemic preconditioning (Bell et al., 2005) and in non-ischemic forms of preconditioning such as tachycardia or exercise (Sánchez et al., 2008). To determine if NOX2 was activated in rat hearts after RIPC we quantified p47, a cytosolic NOX2 subunit recruited to the membrane to activate the catalytic subunit gp91 at the membrane. Figure 2A shows that the ratio of p47/gp91 in a membrane-enriched fraction isolated from hearts increased by 3-fold after RIPC. The oxidation rate of NADPH also increased by 2-fold after RIPC as shown in Figure 2B, showing that NOX2 is active in this membrane fraction.

In parallel experiments we isolated exosomes from the blood of control rats and after RIPC by differential ultracentrifugation. The concentration and size of exosomes were determined by nanoparticle tracking analysis. From the blood of one rat, we typically obtained 200 µL of exosomes with a modal size of 89 ± 12 nm and a concentration of 2 ± 0.9 × 10^10^ particles/mL (N = 3). Western blot analysis of these exosomes showed they contain characteristic proteins such as CD63 and CD81 (Figure 2C). Additionally, exosomes isolated after RIPC were enriched in gp91phox and p47phox (Figure 2D), and had a 3-fold rate of NADPH oxidizing activity compared to controls (Figure 2E). These results show that exosomes isolated after RIPC contain active NOX2.

### 3.3 RIPC increased the mRNAs of Nrf2 and of antioxidant enzymes in the heart

**FIGURE 3:**
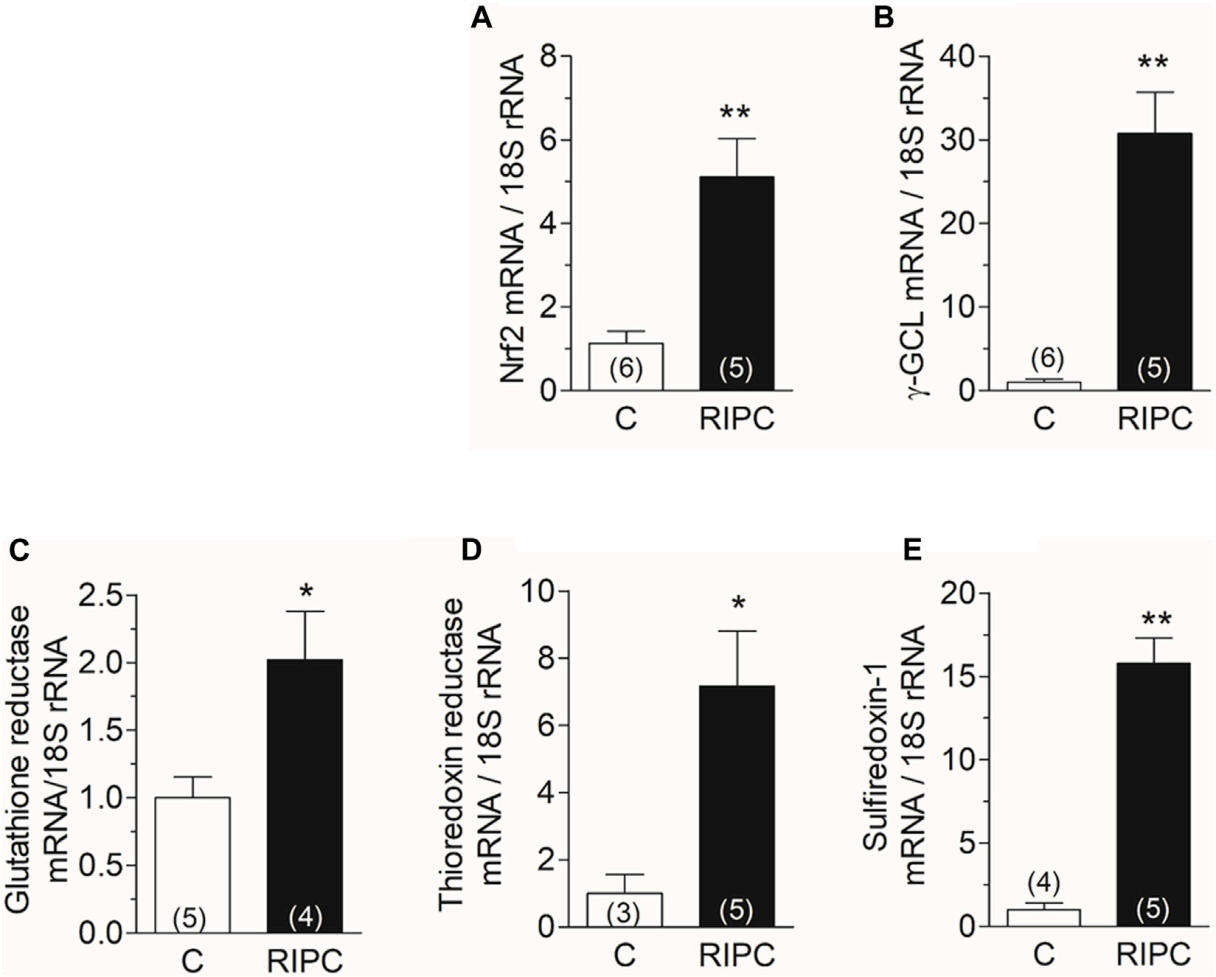
RIPC increases the mRNAs of Nrf2 and of antioxidant enzymes in the heart: Quantification of mRNA by RT-qPCR of **(A)** Nrf2, **(B)** Gamma-glutamyl cysteine ligase, **(C)** Glutathione reductase, **(D)** Thioredoxin reductase, **(E)** Sulfiredoxin-1. Bars show the mean ± SEM of the number of hearts indicated in each bar. Mann Whitney test; *p < 0.05, **p < 0.001.

The activation of NOX2 suggests that ROS are generated in the hearts of rats subjected to RIPC. ROS may activate the antioxidant defenses of the heart. Nrf2 is a transcription factor involved in the transcriptional regulation of several antioxidant enzymes; therefore, we measured by qRT-PCR the mRNA of Nrf2 and of enzymes controlled by this transcription factor. We found that RIPC increased the mRNA content of Nrf2 in the heart (Figure 3A) and also increased the mRNA content of gamma-glutamyl-cysteine ligase, the rate-limiting enzyme in the synthesis of glutathione (Figure 3B), glutathione reductase (Figure 3C), thioredoxin reductase (Figure 3D), and Sulfiredoxin-1 (Figure 3E). These results suggest that Nrf2 and antioxidant enzymes controlled by Nrf2 are upregulated by RIPC.

### 3.4 RIPC increases the antioxidant buffer capacity of the heart

**FIGURE 4:**
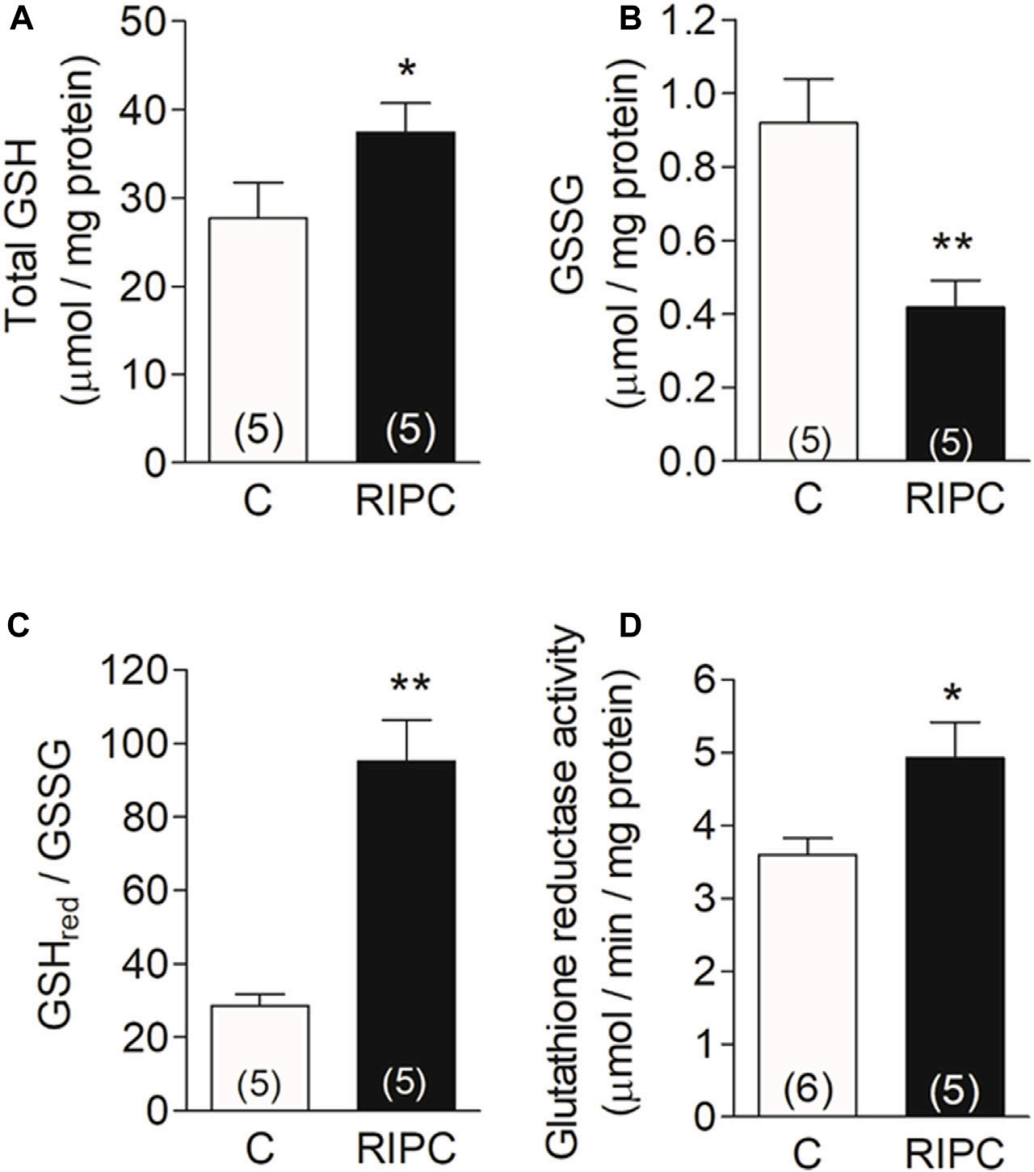
RIPC increases the antioxidant buffer capacity of the heart: **(A)** Total glutathione content (GSH), **(B)** Oxidized glutathione content (GSSG), **(C)** GSHred/GSSG ratio **(D)** Glutathione reductase activity (GR). Bars represent the mean ± SEM of the number of hearts indicated in each bar. Mann Whitney test; *p < 0.05, **p < 0.001.

Glutathione is the primary redox buffer in the heart and changes in concentration or redox state have a deep impact on the antioxidant cell defense. The increase in the mRNAs of gamma-glutamyl-cysteine ligase and of glutathione reductase shown in Figure 3, suggests that RIPC may change the concentration or the equilibrium in the reduced to oxidized form of this redox buffer. We then measured the content of glutathione, the ratio of reduced to oxidized glutathione, and the activity of glutathione reductase in heart homogenates. After RIPC, we observed a 35% increase in total glutathione content (Figure 4A), together with a 55% decrease in GSSG (Figure 4B), resulting in a 70% increase in the GSH/GSSG ratio (Figure 4C). There was also a significant 37% increase in the activity of glutathione reductase (Figure 4D). These changes indicate that the glutathione system is upregulated in the heart and give further support to the notion that RIPC initiates a defensive mechanism against ROS which may play an important role in the defense against the massive amount of ROS produced during reperfusion.

### 3.5 RIPC activates NLRP3 inflammasome in the heart

**FIGURE 5:**
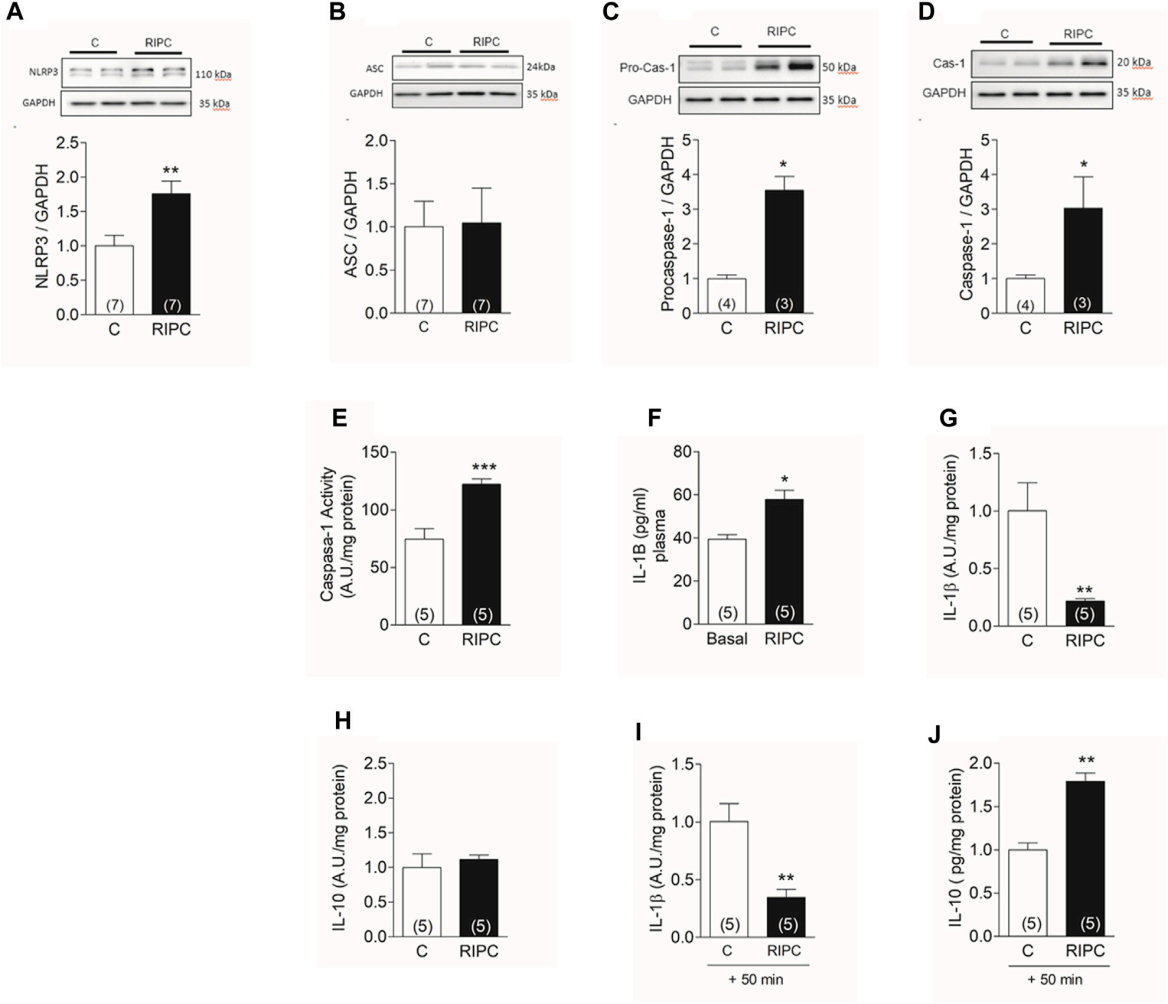
RIPC activates NLRP3 inflammasome in the heart: Representative Western blot determined in heart homogenates and quantification of **(A)** NLRP3, **(B)** ASC, **(C)** Procaspase-1, **(D)** caspase-1. **(E)** caspase −1 activity **(F)** Plasma concentration of IL-1β determined in the same rat before (Basal) and at the end of the preconditioning protocol (RIPC). **(G)** IL-1β in cardiac tissue at the end of RIPC protocol **(H)** IL-10 in cardiac tissue at the end of RIPC protocol **(I)** IL-1β in cardiac tissue after 50 min of perfusion in Langendorff. **(J)** IL-10 in cardiac tissue after 50 min of perfusion in Langendorff. Bars represent the Mean ± SEM of the number of hearts indicated in each bar. Mann Whitney test; *p < 0.05, **p < 0.001.

The abolition of the RIPC protective effect on the infarct size by BAY11-7082 supports a role for NLRP3 inflammasome in the protection. This complex is formed by 3 proteins: the NLRP3 protein, the adapter protein ASC, and pro-caspase-1. Activation of the complex by danger signals cleaves pro-caspase-1. Active caspase-1 processes and releases the inflammatory cytokines IL-1β and IL-18. Both cytokines, especially IL-1β, are upregulated in myocardial infarction and other cardiac pathologies and have a role in the progression to heart failure (Hanna and Frangogiannis, 2020). We found that RIPC produced a 75% increase in the content of NLRP3 protein in heart homogenates (Figure 5A), but no changes in ASC (Figure 5B). There was also a 3.4-fold increase in procaspase (Figure 5C) together with a 3-fold increase in caspase-1 (Figure 5D) determined in western blots of heart homogenates. The enzymatic activity of caspase-1 increased by 63% (Figure 5E). Blood taken before (basal) and at the end of the RIPC protocol showed a significant 30% increase in circulating IL-1β (Figure 5F), indicating that RIPC produced a systemic pro-inflammatory response. To investigate the response of the heart to this pro-inflammatory state, we quantified both, IL-1β and IL-10, an anti-inflammatory cytokine, in cardiac tissue at the end of RIPC and after further perfusion for 50 min. We found that, at the end of RIPC, IL-1β decreased in cardiac tissue (Figure 5G) but IL-10 was unchanged (Figure 5H). After another 50 min IL-1β remained lower than control (Figure 5I) but IL-10 increased by 70% compared to control. This results suggest that the heart activates an anti-inflammatory response that may be responsible, at least in part, for the observed protection.

## 4 Discussion

In this study, we showed that RIPC activates NOX2, upregulates the antioxidant defenses of the heart, and activates the NLRP3 inflammasome which, in turn, initiates an anti-inflammatory response. Both, the upregulation of antioxidant defenses and the anti-inflammatory response confer protection against myocardial IR injury. Inhibition of both, NOX2 or the NLRP3 inflammasome, overrides the protection conferred by RIPC.

To the best of our knowledge, this is the first report of the involvement of NOX2 in RIPC. NOX2 is a complex enzyme made up of two integral membrane subunits, gp91phox and p22phox, and 4 cytosolic subunits (Rac, p40phox, p47phox, p67phox), that are recruited to the membrane when the enzyme is activated. Activation requires Rac’s prenylation and phosphorylation of p47phox to form the active enzyme that transfers electrons from NADPH to oxygen to produce a superoxide anion (Griendling et al., 2000).

Mice hearts KO for NOX2 are not protected by ischemic preconditioning, indicating that NOX2 is necessary for the cardioprotective mechanism (Bell et al., 2005). Also, we have shown that dogs preconditioned by tachycardia or exercise have an increased activity of NOX2 in the heart. Apocynin, a NOX2 inhibitor given before the preconditioning maneuver avoids both, the increase in NOX2 activity and the cardioprotection (Sánchez et al., 2008). At least two reasons can explain the critical role of NOX2 in preconditioning. First, ROS are second messengers that activate several protective pathways (reviewed in Perrelli et al., 2011; Cadenas, 2018). Second, a small amount of ROS generated during preconditioning produces reversible redox modification of critical cysteine residues of key proteins that prevent their irreversible oxidation when challenged with a massive amount of ROS (Yan, 2014). In this regard, we have shown that increased NOX2 activity in exercise or tachycardia preconditioning produces S-glutathionylation of the Ryanodine receptor, the calcium channel of the sarcoplasmic reticulum, turning them resistant to degradation during IR, a fact that may contribute to cardioprotection (Sánchez et al., 2005; Sánchez et al., 2008).

Circulating exosomes isolated from rat’s blood after RIPC contained active NOX2. Exosomes isolated from RIPC animals when infused to naïve hearts, decrease the infarct size to an extent similar to that observed in RIPC (Vicencio et al., 2015; Davidson et al., 2018). It is plausible, therefore, that NOX2-containing exosomes are endocytosed by cardiomyocytes or other cells in the heart, and generate ROS which initiates protective pathways or causes redox modifications of cellular proteins protecting them from irreversible damage. A recent study has shown that exosomes released from macrophages contain functional NOX2. When internalized by damaged axons these exosomes reach the cell body and generate ROS which stimulates the PI3K-pAKT pathway and produces axonal regeneration (Hervera et al., 2018).

The rapid activation of Nrf2 and the transcription of the enzymes involved in the regulation of the glutathione system was a previously unnoticed effect induced by RIPC and most likely plays an important role in cardioprotection. It has been known for a long time the inverse relationship between glutathione content and cardiac infarct size (Singh et al., 1989) and the expression of enzymes catalyzing glutathione synthesis and other glutathione-related antioxidant reactions are under the control of Nrf2 (Ma, 2013).

Reports in the literature indicate that the NLRP3 inflammasome is activated after IR and mediates the damaging effect of IR in the long term. Mice deficient in NLRP3, ASC or caspase-1 have smaller infarcts after IR (Kawaguchi et al., 2011; Liu et al., 2014). Inhibition of NLRP3 inflammasome at the moment of reperfusion has beneficial effects when assessed at 24 h-7 days (Marchetti et al., 2015; Toldo et al., 2016), but no effects are observed in response to acute IR (Jong et al., 2014). In mice subjected to RIPC, the expression of IL-10 is evident after 24 h of reperfusion (Cai et al., 2012).

Our data showed that the NLRP3 inflammasome is rapidly activated by RIPC and the protective effect against IR is evident in less than 2 h after the end of the RIPC protocol. Moreover, the increase in caspase −1 is observed as soon as the preconditioning protocol ends (total duration 40 min) and this activation is critical because its inhibition, via inhibition of the NLRP3 inflammasome, overrides the protection. IL-1β increased in the circulation by the end of the RIPC protocol while decreased in cardiac tissue, and IL-10 increased in cardiac tissue a short time after (around 50 min). IL-10 is an anti-inflammatory cytokine that decreases the generation of other inflammatory cytokines (Moore et al., 2001). We do not know what cells are responsible for the increase in IL-10. Cardiomyocytes produce IL-10 (Stafford et al., 2020) but it can also be produced by endothelial cells or by resident immune cells. The number of endothelial cells in the heart is higher than the number of cardiomyocytes or fibroblasts (Pinto et al., 2015). Also, the number of immune cells is high (Bönner et al., 2012; Zuurbier et al., 2012) and they could contribute to the increase in cytokines or other activities observed in this work.

In conclusion, we showed here that both NOX2 and NLRP3 are needed for the short-term protection mediated by RIPC, probably because they increase the antioxidant defenses of the heart and/or the production of anti-inflammatory cytokines.

## Data Availability

The original contributions presented in the study are included in the article/Supplementary Material, further inquiries can be directed to the corresponding authors.
